# Monochromatic X-ray fluorescence spectroscopy for major and trace element analysis in plant science applications

**DOI:** 10.1007/s11104-025-07984-5

**Published:** 2025-11-13

**Authors:** Chenyu Zhang, Lucas Charrois, Julien Jacquet, Catherine Sirguey, Zewu Chen, Antony van der Ent

**Affiliations:** 1 Laboratory of Genetics, Wageningen University and Research, Wageningen, The Netherlands; 2 Université de Lorraine, INRAE, LSE, F-54000 Nancy, France; 3 Econick SAS, F-54300 Lunéville, France; 4 Z-Spec Inc., East Greenbush, NY United States; 5 Key Laboratory of Biology, Genetics and Breeding of Special Economic Animals and Plants, Ministry of Agriculture and Rural Affairs, Tea Research Institute of the Chinese Academy of Agricultural Sciences, 310008 Hangzhou, China

**Keywords:** Monochromatic X-ray fluorescence, Plant, Element determination, Sample weight, Acquisition time

## Abstract

**Background and aims:**

Determining elemental concentrations in plant tissues is crucial for any study on plant nutrition, physiology, contamination and food safety. However, existing methodologies based on acid digestion of samples coupled to inductively coupled plasma-atomic emission spectrometry (ICP-AES) or plasma-mass spectrometry (ICP-MS) are time-consuming and expensive.

**Methods:**

This study introduces an innovative approach for the rapid and reliable analysis of light, transition, and heavy elements in plant samples  using a novel monochromatic X-ray fluorescence (MXRF) spectrometer.

**Results:**

The MXRF method was tested for the detection of 12 different elements, including light elements (K, Ca), transition metals (Mn, Fe, Co, Ni, Cu, Zn), metalloids (As, Se), and post-transition “heavy” elements (Tl, Pb), covering concentrations from 1 to 10,000 mg·kg^−1^. The limits of detection and quantification ranged from 1.41 to 4.71 mg·kg^−1^. The recovery rates varied from 84.74% to 89.34%, with intraday relative standard deviations (RSD) ≤ 2.31% and inter-day RSD ≤ 4.17%. A method-comparison study using 144 plant samples analysed by both MXRF and ICP-AES showed strong correlations (*R*^*2*^ > 0.87) for K, Ca, Mn, Fe, Co, Ni, Cu, Zn, As, Pb, and TI.

**Conclusions:**

This study demonstrates the reliability of the MXRF technique for the quantification of K, Ca, Mn, Fe, Co, Ni, Cu, Zn, As, Se, Pb, and Tl in plant samples. Given that MXRF can also be applied to the analysis of elemental concentrations in soil and water samples, future research will focus on refining and establishing methodologies for these sample types.

**Supplementary Information:**

The online version contains supplementary material available at 10.1007/s11104-025-07984-5.

## Introduction

Plants take up both essential and toxic elements from the soil and accumulate them in various tissues (Huang et al. 2024). The absence of essential elements in the soil can lead to nutrient deficiencies, resulting in stunted growth or reduced yields (Ahmed et al. 2024). Conversely, high concentrations of toxic elements pose risks to both plants and humans through direct or indirect exposure (Angon et al. 2024). Consequently, assessing elemental concentrations in plants is a critical indicator for farmers, helping to identify soil deficiencies or contamination and enabling necessary adjustments to support healthy plant growth. In addition, the study of plants with abnormally high accumulation of specific elements, refer to as hyperaccumulation, is of significant interest to phytomining and phytoremediation applications (van der Ent et al. 2015).

Several analytical techniques are available for measuring elemental concentrations in plant samples. Among the most used methods are those based on inductively coupled plasma (ICP): inductively coupled plasma-atomic emission spectrometry (ICP-AES) and inductively coupled plasma-mass spectrometry (ICP-MS). These ICP-based techniques require only a small sample quantity (10–300 mg plant material) and are capable of measuring many different elements simultaneously (up to 40 elements) with a dynamic range of at least six orders of magnitude (capable of measuring of 50 parts per billion to several percent mass). ICP-based techniques rely on acid digestion to completely break down sample matrix before analysis of the digestates by ICP-AES/MS analysis. Many elements (notably Si, Cr, Zr, Ti) cannot be accurately determined due to their insolubility during acid digestion, unless hydrofluoric acid or alkaline fusion is used (Pappas 2012). During aerosol generation in the ICP-AES/MS, several processes occur, including ionization, excitation, atomization, vaporization, and desolvation (Bizzi et al. 2017), this poses challenges for hydride-forming elements (As, Sb, Se) and anions (Cl, Br, I). These processes can also introduce matrix effects, which compromise measurement accuracy. Additionally, ‘memory effects’ can arise when elements, especially Hg or Tl, adhere to the sample introduction system and are gradually released over time, leading to falsely elevated readings despite constant initial concentrations (Zhu and Alexandratos 2007). Furthermore, the high cost of equipment and operation (consumables, high-purity argon gas, etc.), along with the required technical expertise, poses additional constraints on the use of ICP-AES/MS analysis (Wilschefski and Baxter 2019).

X-ray fluorescence spectrometry (XRF) is a multi-element analytical technique that enables quantitative analysis by detecting fluorescent X-ray photons emitted from a sample by irradiation using X-rays with energies in the range of 5–50 keV (van der Ent et al. 2018). The XRF signal is unaffected by the chemical environment of the atoms within the sample, which often negates the need for complex sample preparation. This characteristic has rendered (handheld) XRF a widely used tool for rapid on-site detection across various fields, including archaeology, geological exploration, and environmental protection (Pringle et al. 2022; Shackley 2011). In the plant sciences, XRF is particularly useful for the comprehensive characterization of the ionome and for discovery of hyperaccumulator plants in “Herbarium XRF Ionomics” (van der Ent et al. 2019). Elements including Ni, Mn, Co and Zn in herbarium specimens can be measured at a rate of 300 specimens per day, thus enabling mass screening of tens of thousands of herbarium samples (Purwadi et al. 2022, 2021). However, it is worth noting that XRF also has important challenges that can distort the relationship between an analyte’s true concentration and its apparent measured concentration. Matrix effects, arising from both physical and chemical properties, are particularly problematic (Endriss et al. 2024; Tavares et al. 2023). Physically, variations in sample density, particle size, and water content can alter X-ray absorption and scattering, thereby reducing reproducibility and quantitative accuracy (Ge et al. 2005). Furthermore, differences in structure and composition can elevate background noise and diminish sensitivity (Criss 1976). In addition, spectral interferences occur from multiple sources, including characteristic X-ray peaks, escape peaks, overlapping peaks, and electronic noise, which are all superimposed on the continuous background spectrum. Such interferences further complicate accurate spectral processing. A persistent challenge, therefore, lies in reliably extracting the net peak area of characteristic X-ray fluorescence spectra from full-spectrum data (Li et al. 2020). Moreover, quantification of light elements (e.g., Mg, Al, Si, P, S, Cl) remains particularly difficult, as photoelectric excitation in these cases predominantly relaxes via the emission of Auger electrons. This process greatly reduces fluorescence yields and, consequently, limits the achievable signal-to-noise ratio (Kikongi et al. 2017).

Desktop and handheld XRF instruments typically rely on polychromatic excitation, whereby the X-ray source tube produces an incident beam for excitation with an arc-like shape of the Bremsstrahlung background with fluorescent peaks arising above this background. Energy-dispersive XRF instruments use silicon drift detectors (SDD) that have a resolution of ~ 135 eV (at Mn Kα at 5.900 keV) which is not sufficient to resolve certain line overlaps, including the Lα_1_ line of Pb (at 10.551 keV) with the Kα_1_ of As (at 10.543 keV) and the Fe Kβ_1_ line (at 7.058 keV) with the Co Kα_1_ line (6.9303 keV) (Purwadi et al. 2021). This means that analysing As in the presence of high prevailing Pb or Co in the presence of high prevailing of Fe presents a problem. Monochromatic XRF instrument (MXRF) has doubly curved crystals to produce a monochromatic excitation source (Chen and Wittry 1998). This results in a dramatically lower background (approximately 100 times) with a signal to noise ratio (dependent on the concentration of an analyte) than polychromatic XRF (Chen et al. 2008). Practically, this translates to much lower limits of the detection (LODs), improved spectral deconvolution, and ease of quantification using a Fundamental Parameters approach (Kawai et al. 2019). For instance, Johnson-Restrepo et al. (2025) reported that the LODs of Cd (0.04 mg·kg^−1^) for the MXRF instruments were up to 100 times lower than polychromatic XRF (5 mg·kg^−1^). The highest sensitivity in XRF is achieved when the excitation energy is closest to the absorption edge of the element to be analysed. Since only a single monochromatic energy can be produced using a crystal monochromator tube source, there is a trade-off between the ability to measure many elements of the Periodic Table and analytical performance. Moreover, it must be also noted that the contribution of X-ray scattering to XRF spectra with monochromatic excitation is considerably lower than using polychromatic excitation (Hodoroaba et al. 2011). The reduction in flux necessitates either longer acquisition times or the application of higher tube voltage and current settings. For certain elements, this trade-off may lead to reduced sensitivity compared with polychromatic XRF systems. The company Z-Spec Inc. has recently brought MXRF instruments on the market with distinct excitation energies to enable analysis of light elements (Z = 11 Na to 20 Ca using 4.51 keV excitation, E-lite instrument), medium range elements (Z = 15 P to 39 Y using the K-lines and up to Z = 92 (U) via the L-lines using 17.48 keV excitation, JP500 instrument), and high range elements (as JP500, but extending K-lines to include Z = 42 Mo to Z = 53 I using 35 keV excitation, E-Max instrument). This study focuses on the JP500 instrument, which has an optimum sensitivity for elements Cu–Se and Hg-Tl-Pb with LODs reported to be 0.009–0.025 mg·kg^−1^ (https://zspecinc.com/jp500).

Two types of calibrations can be employed to convert the raw data (counts per second at energies) of the XRF instrument measurement to elemental concentrations: Empirical Calibration (EC) and Fundamental Parameters (FP). Whereas EC involves modelling the quantitative correlation between measured net intensities and concentrations of a set of standards with a similar matrix as the samples, FP is a physics equation that calculates fluorescent radiation of elements in a sample to derive elemental concentration. The EC approach is simple and can be undertaken by the user of the XRF instrument. It entails using linear regression formulas for each element to ‘correct’ XRF readings from the instrument. Fundamental Parameters is often proprietary firmware not accessible to the user/owner, and frequent used for plant bulk samples (Rousseau 2013) and herbarium XRF ionomics (Purwadi et al. 2022, 2021). It necessitates that all experimental parameters and sample characteristics are known, which is straight forward for the former, but not for the latter as bulk composition and density are sample dependent variables. Either assumptions are made, using cellulose (C_6_H_10_O_5_) with a density of 1.4 g·cm^−3^ as a proxy for ‘typical plant material’ and a thickness meeting the ‘infinite thickness’ requirement. Plant material consists largely of cellulose and hence the bulk composition is C, H, O, and is transparent to X-rays in the 5–50 keV range used in XRF. As a consequence, it does not fulfil the ‘infinite thickness’ requirement in the FP equation, unless at least 5 mm thickness is used at the 17.48 keV incident energy of the JP500 instrument (or more importantly the maximum escape depth of fluorescent X-rays < 15 keV). It is possible to estimate the sample thickness by using the Compton Scatter of the sample and use that in the Fundamental Parameters.

A previous study has established a quantification method for As, Hg, Tl, and Pb in human tissues using MXRF implemented in the JP500 instrument (Wu et al. 2023). Furthermore, an earlier study examined the performance of the Z-Spec E-lite instrument for analysis of light elements, covering Na-Ca (Kahlon et al. 2024). It systematically evaluated different sample preparation methods, repeatability, and measurement times, and showed that MXRF method delivers rapid, non-destructive, and extraction-free analysis that strongly correlates with ICP-MS acquired data. The current study aims to develop a method for accurate and reliable analysis of 12 elements: light elements (K, Ca), transition metals (Mn, Fe, Co, Ni, Cu, Zn), metalloids (As, Se), and post-transition elements (Tl, Pb) in plant samples using MXRF.

## Materials and methods

### Instrumentation and operational procedures

**Fig. 1:**
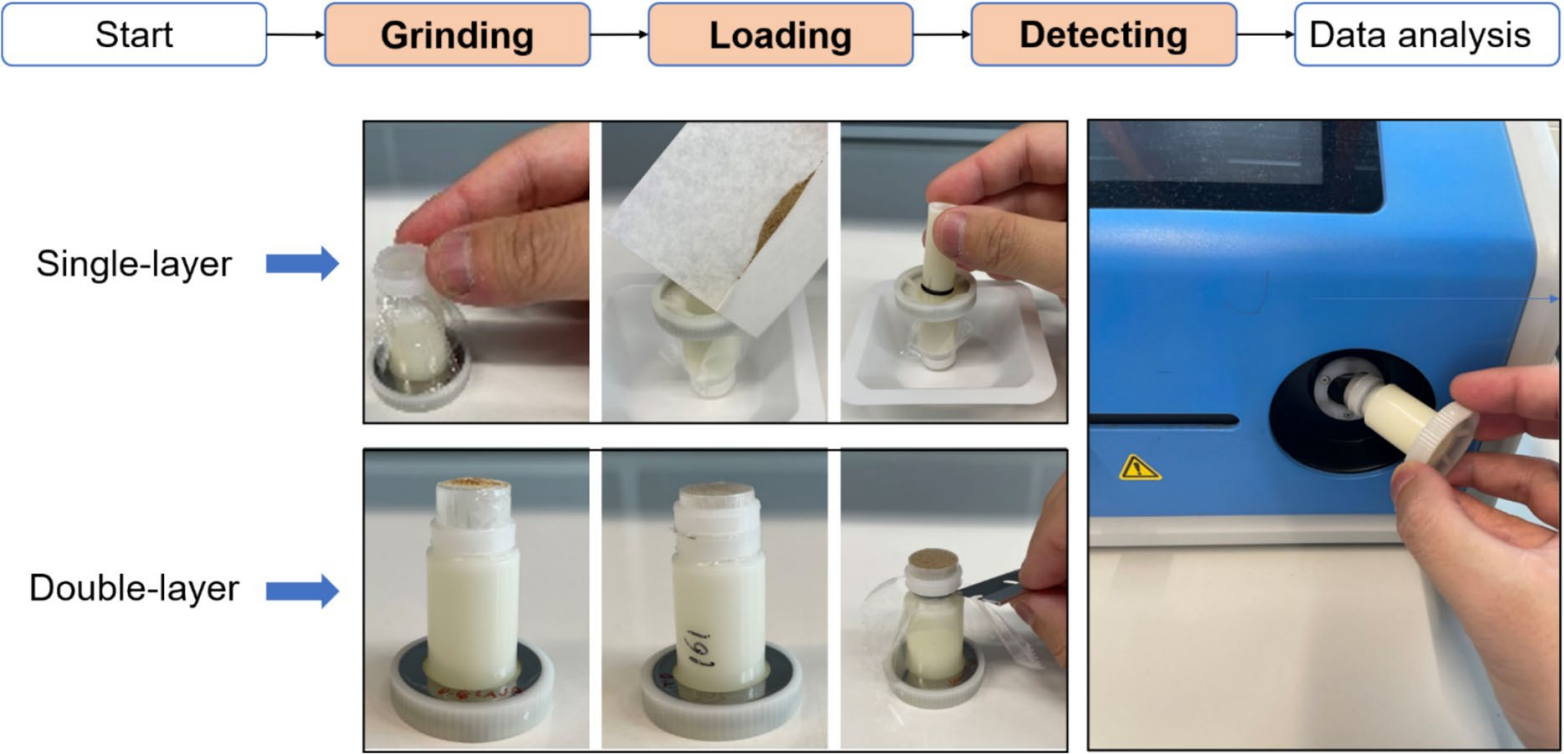
General workflow of elemental analysis of plant samples using MXRF spectrometer

The monochromatic X-ray fluorescence (MXRF) analyser (model JP500) and 11 mm diameter custom sample holder cups were purchased from Z-Spec Inc. (New York, USA). The polypropylene film was purchased from Chemplex Industries Inc. (New York, USA). The tube target material is Mo, operating voltage is 50 kV and current is 0.5 mA; the detector type is Fast Silicon drift detectors (SDD) of 0.5 mm thickness with a spectral resolution of 138 eV at 5.9 keV, the measurement atmosphere is air and the beam spot size is 3 mm. As shown in Fig. 1, the general workflow of MXRF spectrometer includes three steps: grinding, loading, and detecting, which largely reduce the pretreatment time and manpower comparing to the ICP-based method. The dried plant material was powdered using a batch mill (Tube Mill 100 control, IKA, Germany) operated at 20,000 rpm for 1 min. The ground material was subsequently sieved through a 100-mesh sieve (150 μm opening size, Gilson), and the resulting fine powder was used for measurement. Since the MXRF spectrometer provides two detection modes for thin (≤ 20 mg) and thick samples (100–300 mg), both were evaluated separately. The key parameters of the analytical procedure included sample weight and measurement time. A 3^2^ factorial design with the spiked cellulose samples at 1000 mg·kg^−1^ for each of the 12 elements was used to determine the optimum analytical conditions for each mode. Phosphorus (P), sulfur (S), and chlorine (Cl) were excluded from this study; therefore, the described methodology could not be applied to their measurement. For the standard MXRF analysis, 100 mg of material was weighed and transferred into a holder, which was then covered with a 6.0 µm polypropylene film (Chemplex Industries Inc). For small sample analysis, the holder was first covered with a 6 µm polypropylene film, onto which 20 mg of powder was placed. This was then covered with an additional 6.0 µm polypropylene film. The analysis time was set at either 30, 60, 120 s.

### Elemental spike to determine accuracy, precision, LODs and LOQs

For the spiking assay, cellulose powder (310,697, Sigma-Aldrich, Missouri, USA) was used as a blank, as the concentrations of the 12 target elements were negligible. Twelve water-soluble salts, representing each element, were prepared using deionized water. These salts included sodium arsenate dibasic heptahydrate (As), calcium chloride dihydrate (Ca), cobalt chloride hexahydrate (Co), copper (II) sulfate pentahydrate (Cu), iron (II) sulfate heptahydrate (Fe), potassium chloride (K), manganese (II) sulfate tetrahydrate (Mn), nickel chloride hexahydrate (Ni), lead acetate trihydrate (Pb), sodium selenate (Se), thallium nitrate (Tl), and zinc acetate dihydrate (Zn). These reagents were sourced from Merck (Darmstadt, Germany) and Sigma-Aldrich (Missouri, USA).

To minimize spectral overlap, the 12 elements were categorized into three groups, particularly to avoid interferences among As (Kα, 10.543 keV), Tl (Lα, 10.269 keV), and Pb (Lα, 10.551 keV). A stock solution of each of the 12 elements was prepared at a concentration of 50 g·L^−1^ **(**Table S1**)**. A specified volumes of the stock solution was added to 1 g of cellulose to create a mixed standard (Table S2), to obtain the concentrations described below. Calibration curves were established by spiking the blank cellulose with final concentrations of 1, 5, 10, 50, 100, 500, 1000, 5000, and 10,000 mg·kg^−1^ of each element. The measured concentrations obtained by MXRF were plotted against the spiked levels to produce regression curves. This aimed to compare “real concentrations” in spiked model samples with those obtained by the MXRF to test the internal calibration through the determination of the accuracy and the precision. For each spiking concentration, multiple independent cellulose blanks were prepared at each spiking concentration and analysed once. The limit of detection (LOD) and limit of quantification (LOQ) values were calculated according to the ICHQ2(R2) (Agency 2023), and the formula were as follows: $LOD=\frac{3.3\times \sigma }{S}$ and $LOQ=\frac{10\times \sigma }{S}$. σ is the standard deviation of the blank and S was the slope of the calibration curve. Accuracy and precision were assessed by spiking a blank with 10 and 1000 mg·kg^−1^ mixed standards. Recovery was calculated by dividing the measured concentration by the theoretical concentration. The relative standard deviation (RSD) was determined by dividing the inter-day and intraday standard deviations by the mean value obtained from six replicates.

### Empirical comparison between MXRF and ICP-AES

Plant material samples were chosen to cover a range of elemental concentrations for macro and trace elements and full details on the nature and origin of the samples is provided in Table S3. A total of 144 plant samples were randomly selected and tested twice using both the miniaturized method (batch 1) and the standard method (batch 2). To further validate the reliability of MXRF measurements, 144 plant samples were randomly selected for analysis. Each sample was first measured using MXRF, then retrieved and subsequently analysed using ICP-AES. For each element, regression analysis was conducted by comparing the two datasets, and the corresponding Pearson correlation coefficients (*R*^*2*^) were calculated. In addition, Bland–Altman plots were used to assess the agreement between MXRF and ICP-AES. The plant material was dried in a drying oven at 60℃ for a minimum of 48 h. The dried sample was initially ground using a batch mill (Tube Mill 100 control, IKA, Germany) at 20,000 rpm for 1 min. Subsequently, approximately 0.5 g of the sample was further ground with a ball mill at HZ for 60 s. After completing the MXRF analysis, as described below, plant material samples were acid digested for ICP-AES analysis. First, 50 mg sample was weighted and transferred into 15 mL polypropylene tubes and the samples were pre-digested for 16 h with 1 mL HNO_3_ (70%) and 2 mL H_2_O_2_ (30%) following the NF ISO 11464. Then, the samples were digested in a block heater (DigiPREP Jr, SCP SCIENCE) for a 180 min programme (ramping up and hold at 95℃). The samples were brough to a final volume of 10 mL with ultrapure water and filtered through 0.45 µm syringe filters before analysis with ICP-AES using a Thermo Scientific iCAP PRO Duo X instrument in axial or radial mode as required.

### Characterization of the influence of sample fineness and density

To assess sample fineness, 0.1 g of an unground sample (with a particle size > 2 mm) was tested. Then, the tested sample was ground into a fine powder (150 µm) and retested. For sample density determination, 0.1 g of the sample was placed into the sample cup. Initially, the plunger was lightly inserted into the sample cup for preliminary detection, positioning the top of the plunger 7 cm from the film. Subsequently, the plunger was firmly inserted into the sample cup for detection, reducing the distance to 4 cm from the film. Comparing the results of these two tests clarifies the effects of sample fineness and density on measurement stability.

### Statistical analyses

The Student's *t*-test and two-way ANOVA with Tukey's multiple comparisons test in parametric conditions were employed to evaluate difference between each pair of means using GraphPad Prism (Version 8.3.0), with *P* < 0.05 considered statistically significant. All data are presented as the mean values ± standard deviation, based on three to nine independent replicates.

## Results

### Optimization of analytic procedures for MXRF analysis

To evaluate recovery rates under different conditions, a mixed spiked sample (1000 mg·kg^−1^) was used across various sample weights and detection times in thin sample (double-layer) and thick sample (single-layer) modes. Average recoveries across 12 elements were calculated to optimize the two major factors affecting MXRF performance (measurement time and sample weight), as the method is designed for simultaneous multi-element detection. The result showed that the average recoveries (*i.e.,* known values *vs*. measured values) of 12 elements ranged from 12.47% to 89.61% for thin sample analysis **(**Table 1**)**, while the average recovery of 12 elements ranged from 85.17% to 116.90% for thick sample analysis **(**Table 2**)**. A two-way ANOVA was performed to assess the effects of sample weight and measurement time on average recovery. The analysis revealed that sample weight had a significant effect (*P* < 0.05) on the recovery of thin samples (Table S3), while no statistically significant differences were observed for thick samples (Table S4). To further clarify the relative influence of the two factors, a range analysis was conducted. The results show that the optimal sample weights for thin and thick samples were 20 mg and 100 mg, respectively, and the optimal measurement time was 30 s (Table S5, S6). Subsequent experiments were therefore conducted using these optimized measurement conditions.

**Table 1:** Factorial design and average recovery for thin samples (sample weight < 20 mg)

Combination	Factor		Average recovery (%)
	A	B	
1	1	1	20.51
2	1	2	18.77
3	1	3	12.47
4	2	1	55.93
5	2	2	43.53
6	2	3	59.84
7	3	1	89.61
8	3	2	87.00
9	3	3	85.94

*Factor A) was sample weight, which included three levels: 1) 5 mg, 2) 10 mg, and 3) 20 mg; factor B was measurement time, which included three levels: 1) 30 s, 2) 60 s, and 3) 100 s*

**Table 2:** Factorial design and average recovery for thick samples (sample weight > 100 mg)

Combination	Factor		Average recovery (%)
	A	B	
1	1	1	95.96
2	1	2	106.1
3	1	3	116.9
4	2	1	110.9
5	2	2	93.35
6	2	3	88.4
7	3	1	95.38
8	3	2	85.17
9	3	3	95.44

*Factor A) was sample weight, which included three levels: 1) 100 mg, 2) 200 mg, and 3) 300 mg; factor B was measurement time, which included three levels: 1) 30 s, 2) 60 s, and 3) 100 s*

### Effect of sample fineness and packing density for MXRF analysis

**Fig. 2:**
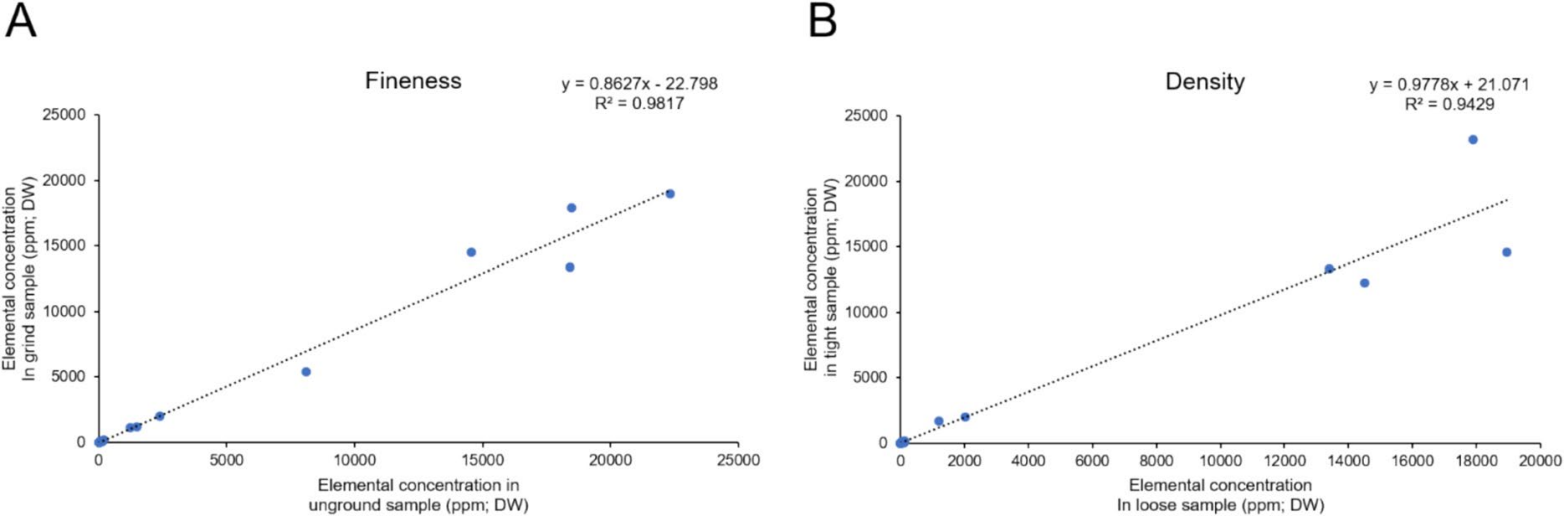
Effect of sample fineness and density on measurement stability. Correlation analyses were performed for sample fineness (A) and packing density (B). The analysed elements include K, Ca, Mn, Fe, Ni, Cu, Zn, As, Se, and Co. Each point represents one element, based on measurements from samples with varying degrees of fineness and density. R^2^ represents the Pearson correlation coefficient

To assess the influence of physical preparation parameters on XRF measurement repeatability, two key parameters were examined: sample fineness and packing density. A strong correlation was observed between ground and unground samples (R^2^ of 0.98; Fig. 2a). Likewise, variations in bulk density and compaction showed modest influences, with measurements under loosely and tightly packed conditions yielding a high degree of consistency (R^2^ of 0.94; Fig. 2b).

### Linearity and range of the MXRF analysis

We spiked cellulose powder with 12 elements across a wide range of concentrations to evaluate the calibration performance of each element under both detection modes. Good linearity (*R*^*2*^ > 0.99) was observed for all elements within the concentration range of 1 mg·kg^−1^ to 10,000 mg·kg^−1^. The LODs and LOQs for the thin sample ranged from 0.01 mg·kg^−1^ to 17.28 mg·kg^−1^ and 0.01 mg·kg^−1^ to 52.36 mg·kg^−1^, respectively **(**Table 3**)**; In the thick sample mode, the LOD and LOQ ranged from 0.01 mg·kg^−1^ to 18.38 mg·kg^−1^ and 0.01 mg·kg^−1^ to 55.71 mg·kg^−1^, respectively **(**Table 4**)**. The highest LOD and LOQ values were observed for Fe and Ni in both modes, suggesting a potential systematic bias in MXRF detection for these elements.

**Table 3:** Parameters of working range for thin samples in MXRF spectrometer

Element	Linear range (mg·kg^−1^)	Regression equation	Correlation coefficient, *R*^*2*^	LOD (mg·kg^−1^)	LOQ (mg·kg^−1^)
As	1–10,000	y = 0.736x + 10.611	1	0.99	2.99
Ca	1–10,000	y = 0.9323x + 31.611	1	0.74	2.25
Co	1–10,000	y = 1.0947x—30.529	0.9998	0.43	0.51
Cu	1–10,000	y = 1.0677x—2.896	0.9999	0.09	0.28
Fe	1–10,000	y = 1.4475x—12.784	1	16.02	48.53
K	1–10,000	y = 0.8967x—20.833	1	3.96	11.99
Mn	1–10,000	y = 1.3837x—42.101	0.9998	0.26	0.80
Ni	1–10,000	y = 1.3101x—42.435	0.9999	17.28	52.36
Pb	1–10,000	y = 1.1036x—75.032	0.9989	0.01	0.4
Se	1–10,000	y = 1.0331x + 70.103	0.9992	0.05	0.15
TI	1–10,000	y = 1.2026x—79.161	0.9992	0.01	0.04
Zn	1–10,000	y = 1.0174x—18.828	1	0.01	0.01

**Table 4:** Parameters of working range for thick samples in MXRF spectrometer

Element	Linear range (mg·kg^−1^)	Regression equation	Correlation coefficient, *R*^*2*^	LOD (mg·kg^−1^)	LOQ (mg·kg^−1^)
As	1–10,000	y = 0.7293x + 18.822	0.9999	1.00	3.02
Ca	1–10,000	y = 1.1253x—2.6888	0.9999	0.62	1.87
Co	1–10,000	y = 0.9052x—0.3746	1	0.01	0.11
Cu	1–10,000	y = 0.9029x—18.157	0.9998	0.11	0.33
Fe	1–10,000	y = 1.4774x—35.982	0.9999	15.69	48.54
K	1–10,000	y = 1.0528x + 28.411	0.9997	3.37	10.21
Mn	1–10,000	y = 1.4055x—63.925	0.9997	0.26	0.78
Ni	1–10,000	y = 1.2314x—46.834	0.9998	18.38	55.71
Pb	1–10,000	y = 1.1499x—35.142	0.9998	0.21	0.51
Se	1–10,000	y = 0.7528x + 39.007	0.9994	0.07	0.20
TI	1–10,000	y = 0.9134x—42.959	0.9997	0.02	0.05
Zn	1–10,000	y = 0.8049x—19.551	0.9999	0.01	0.01

### Accuracy, stability, and precision of the MXRF analysis

The accuracy and precision of the MXRF for 12 elements were first evaluated using two spiked samples (10 and 1,000 mg·kg^−1^), and by calculating intra- and inter-day relative standard deviations (RSDs). For thin sample, recoveries of all elements ranged from 77.39% to 121.86%, with intra-day RSDs ≤ 17.48% and inter-day RSDs ≤ 15.17% **(**Table 5**)**. Among them, relatively high RSD values (> 10%) were observed for Co (10.73%) and Mn (15.17%). For thick sample, recoveries of 12 elements ranged from 72.66% to 128.25%, with intra-day RSDs ≤ 6.80% and inter-day RSDs ≤ 9.98% **(**Table 6**)**, with the highest RSD recorded for Ca.

**Table 5:** Accuracy and precision of elemental analysis for thin samples in MXRF spectrometer

Element	Spiked concentration (mg·kg^−1^)	Mean recovery (%)	Intra-day RSD% (*n* = 6)	Inter-day RSD% (*n* = 6)
As	10	80.86	1.57	2.31
	1000	77.39	3.35	5.07
Ca	10	116.55	3.69	5.05
	1000	102.42	3.56	2.52
Co	10	117	3.51	5.25
	1000	104.93	15.2	10.73
Cu	10	119.8	2.2	3.81
	1000	100.2	2.79	4.87
Fe	10	116.16	9.02	6.65
	1000	118.74	1.61	2.16
K	10	87.58	2.85	3.46
	1000	86.73	5.45	4.38
Mn	10	102.86	17.48	15.17
	1000	121.86	1.83	3.69
Ni	10	109.97	3.46	9.8
	1000	103.64	1.21	2.77
Pb	10	114.04	6.05	4.53
	1000	118.15	0.45	3.09
Se	10	97.52	2.33	2.5
	1000	112.57	2.32	2.15
TI	10	113.73	0.04	0.46
	1000	88.44	3.12	6.35
Zn	10	92.9	8.38	4.4
	1000	83.37	1.73	3.22

**Table 6:** Accuracy and precision of elemental analysis for thick samples in MXRF spectrometer

Element	Spiked concentration (mg·kg^−1^)	Mean recovery (%)	Intra-day RSD% (*n* = 6)	Inter-day RSD% (*n* = 6)
As	10	91.05	5.7	4.81
	1000	77.79	2.62	2.16
Ca	10	111.34	6.8	9.98
	1000	104.8	4.05	2.93
Co	10	80.16	4.49	5.14
	1000	92.2	2.61	7.59
Cu	10	113.98	4.27	3.48
	1000	77.24	1.73	1.42
Fe	10	103.72	4.91	4.02
	1000	128.25	3.47	3.36
K	10	119.57	4.74	4.07
	1000	95.29	2.29	2.18
Mn	10	116.62	0.99	6.91
	1000	112.23	3.37	3.71
Ni	10	93.7	0.62	3.54
	1000	104	2.04	2.27
Pb	10	112.12	1.07	0.82
	1000	94.73	4.51	3.47
Se	10	75.08	4.2	8.84
	1000	91.29	1.79	1.48
TI	10	73.95	0.94	3.81
	1000	73.91	0.49	4.19
Zn	10	79.63	5.01	4.6
	1000	72.66	1.1	1.63

To further validate accuracy and stability, recoveries were determined for three NIST-certified reference materials (CRMs: 1515, 1547, and 1570a), which included certified values for all elements except Tl. For thin sample, mean recoveries across all elements ranged from 82 to 114%, with an overall average of 98% **(**Table 7**)**. For thick sample, mean recoveries ranged from 90 to 112%, with an overall average of 103% **(**Table 8**)**. For both modes, the coefficients of variation (CVs) were consistently < 10%, indicating stable recoveries across the three CRMs. With respect to accuracy, MXRF achieved average recoveries within 10% of the certified values for K, Ca, Mn, Co, Ni, Cu, Zn, As, Se, and Pb, while Fe showed slightly larger deviations (10–12%).

**Table 7:** Accuracy and stability of elemental analysis for the 3 NIST CRMs from MXRF spectrometer, using thin sample mode

	K	Ca	Mn	Fe	Co	Ni	Cu	Zn	As	Se	TI	Pb
**NIST 1515**												
Certified value (mg·kg^−1^)	16,100.00	15,260.00	54.00	80.00	0.09	0.91	5.64	12.50	0.04	0.05	N/A	0.47
Mean result by MXRF (mg·kg^−1^)	15,700.00	15,065.00	50.12	75.09	0.09	0.96	4.92	11.90	0.04	0.06	0.13	0.50
Mean recovery (%)	97.52	98.72	92.81	83.86	96.67	105.38	87.15	95.16	92.11	110.00	N/A	106.38
**NIST 1547**												
Certified value (mg·kg^−1^)	24,300.00	15,600.00	98.00	220.00	0.07	0.69	3.70	17.90	0.06	0.12	N/A	0.87
Mean result by MXRF (mg·kg^−1^)	21,895.00	14,050.00	85.37	179.86	0.08	0.76	3.93	18.84	0.07	0.11	0.06	0.92
Mean recovery (%)	90.10	90.06	87.11	81.75	114.29	109.42	106.08	105.22	108.33	91.67	N/A	105.75
**NIST 1570a**												
Certified value (mg·kg^−1^)	29,030.00	15,270.00	75.90	N/A	0.39	2.14	12.20	82.00	0.07	0.12	N/A	0.20
Mean result by MXRF (mg·kg^−1^)	27,785.00	14,525.00	76.34	280.79	0.38	2.35	11.39	81.79	0.06	0.13	0.03	0.19
Mean recovery (%)	95.71	95.12	100.58	N/A	98.21	109.81	93.36	99.74	91.77	106.84	N/A	97.50
**CRM summary**												
Average recovery of 3 NIST CRMs (%)	94.44	94.63	93.50	92.81	103.06	108.20	95.53	100.04	97.40	902.84	N/A	103.21
CV (%)	3.34	3.75	5.90	1.27	7.73	1.85	8.25	4.11	7.93	7.78	N/A	3.92

*N/A indicates that no certified concentration is available for the corresponding element in the CRMs*

**Table 8:** Accuracy and stability of elemental analysis for the 3 NIST CRMs from MXRF spectrometer, using thick sample mode

	K	Ca	Mn	Fe	Co	Ni	Cu	Zn	As	Se	TI	Pb
**NIST 1515**												
Certified value (mg·kg^−1^)	16,100.00	15,260.00	54.00	80.00	0.09	0.91	5.64	12.50	0.04	0.05	N/A	0.47
Mean result by MXRF (mg·kg^−1^)	17,785.00	16,700.00	59.93	89.18	0.09	0.95	5.46	13.09	0.04	0.06	0.14	0.44
Mean recovery (%)	110.47	109.44	110.97	111.48	97.78	103.85	96.72	104.72	105.26	110.00	N/A	93.62
**NIST 1547**												
Certified value (mg·kg^−1^)	24,300.00	15,600.00	98.00	220.00	0.07	0.69	3.70	17.90	0.06	0.12	N/A	0.87
Mean result by MXRF (mg·kg^−1^)	26,765.00	16,200.00	107.62	226.69	0.07	0.76	3.69	19.01	0.07	0.13	0.06	0.78
Mean recovery (%)	110.14	103.85	109.82	103.04	94.29	109.42	99.59	106.20	108.33	108.33	N/A	89.66
**NIST 1570a**												
Certified value (mg·kg^−1^	29,030.00	15,270.00	75.90	N/A	0.39	2.14	12.20	82.00	0.07	0.12	N/A	0.20
Mean result by MXRF (mg·kg^−1^)	29,840.00	16,165.00	77.29	289.07	0.39	2.06	11.04	84.21	0.07	0.12	0.04	0.21
Mean recovery (%)	102.79	105.86	101.83	N/A	98.97	96.26	90.49	102.70	95.59	102.56	N/A	105.00
**CRM summary**												
Average recovery of 3 NIST CRMs (%)	107.80	106.38	107.54	107.26	97.01	103.18	95.60	104.54	103.06	106.97	N/A	96.09
CV (%)	3.29	2.17	3.78	3.93	2.05	5.23	3.97	1.37	5.27	2.98	N/A	6.77

*N/A indicates that no certified concentration is available for the corresponding element in the CRMs*

### Empirical comparison between MXRF and ICP-AES

**Fig. 3:**
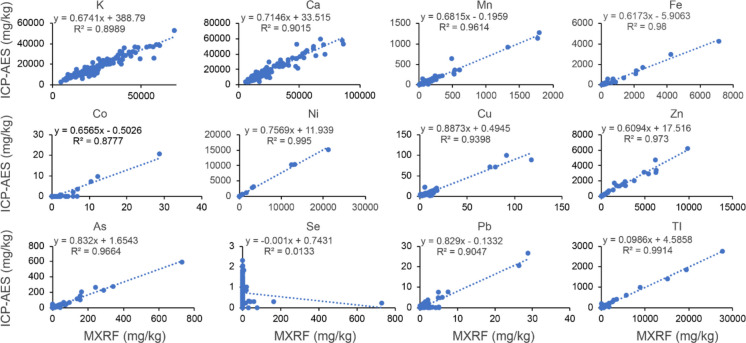
Comparison of concentrations of 12 elements measured by ICP-AES and MXRF. Scatter plots are shown for potassium (K), calcium (Ca), manganese (Mn), iron (Fe), cobalt (Co), nickel (Ni), copper (Cu), zinc (Zn), arsenic (As), selenium (Se), lead (Pb), and thallium (Tl). The x-axis represents MXRF measurements, and the y-axis represents ICP-AES measurements. Each plot includes a fitted linear regression line (dashed blue), with the regression equation and coefficient of determination (R^2^) displayed in the legend

A total of 144 plant samples were measured using both MXRF and ICP-AES, and the results were compared by linear regression **(**Fig. 3**)**. For all 12 elements, MXRF yielded higher mean values than ICP-AES. Significant positive correlations were observed for all elements (*P* < 0.0001; *R*^*2*^ ≥ 0.88), except Se, which exhibited poor correlation (*R*^*2*^ < 0.02). This discrepancy was primarily due to the very low concentrations of Se, with 12 out of 144 samples falling below the ICP-AES LOQ (0.1 mg·kg^−1^). In addition, Tl concentrations measured by MXRF were approximately ten-fold higher than those obtained by ICP-AES.

**Fig. 4:**
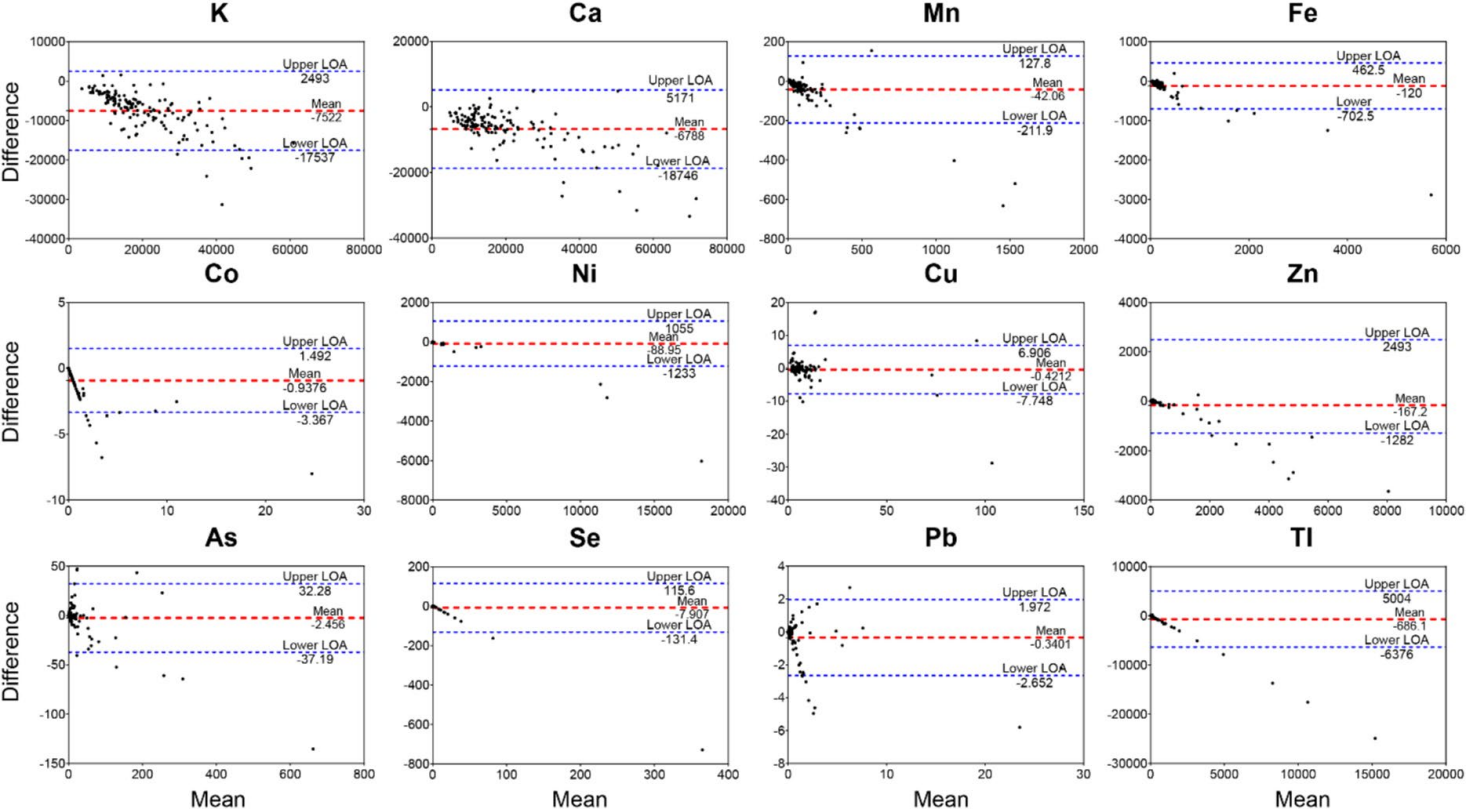
The Bland–Altman plot of concentrations of 12 elements measured by ICP-AES. Plots are shown for potassium (K), calcium (Ca), manganese (Mn), iron (Fe), cobalt (Co), nickel (Ni), copper (Cu), zinc (Zn), arsenic (As), selenium (Se), lead (Pb), and thallium (Tl). The x-axis represents the mean of the two measurements (MXRF and ICP-AES), while the y-axis shows the difference between them. The dashed red line indicates the mean difference (bias), and the dashed blue lines indicate the limits of agreement (± 1.96 standard deviations)

Consistent with the regression analysis, Bland–Altman plots using untransformed data **(**Fig. 4**)** revealed a systematic bias between the two methods, with MXRF consistently reporting higher values (mean difference < 0 mg·kg^−1^). The mean differences were largest for K, Ca, Fe, Zn, and Tl (above 100 mg·kg^−1^); moderate for Mn and Ni (< 100 mg·kg^−1^); and minimal for Co, Cu, As, Se, and Pb (< 10 mg·kg^−1^).

## Discussion

The MXRF technique, as implemented in Z-Spec Inc. instruments, relies on monochromatized excitation combined with the FP method (Chen et al. 2008). Applying the FP method requires conditions including homogeneous and flat samples (Sherman 1955; Shiraiwa and Fujino 1966). A significant challenge involves uncertainties in the mass absorption coefficients and fluorescence yields of individual elements in the sample matrix (Akbulut 2014). In this study, we used cellulose powder, a homogeneous, fine, and clean material, not only to optimize operational parameters but also to evaluate the precision and accuracy of each element. Our results revealed that the sample amount and measurement time did not significantly affect recovery using the single-layer method, which contrasts with a recent study (Wu et al. 2023). This discrepancy may stem from differences in matrix type: we used cellulose powder as a blank matrix, whereas Wu et al. (2023) used pork liver.

When applying the double-layer method, we observed that sample amounts below 20 mg caused considerable variation in recovery, whereas measurement time itself had no important effect. The reduced recovery is most likely attributable to inhomogeneous sample distribution within the holder. This effect is more pronounced in the JP500 instrument, which operates at a relatively high incident energy (17.48 keV). Under these conditions, the matrix exhibits low X-ray absorption, thereby amplifying the impact of uneven sample packing. In contrast, the low-energy E-lite instrument (4.51 keV) is less affected by this issue and can reliably analyse quantities as low as < 5 mg (Kahlon et al. 2024). Consequently, a sample mass of 100 mg provided more stable and higher recovery values, even though such a mass does not constitute an infinitely thick sample for XRF analysis. For an “infinitely thick” sample, the increase in sample thickness has no further effect on the apparent concentration of the element. This is because an increase in mass thickness (sample mass per unit area) does not significantly affect the emitted intensity (Marguí et al. 2009; Zhou et al. 2022). In contrast, for samples of intermediate thickness, changes in mass can lead to variations in the measured fluorescence intensity. In our study, light elements (e.g., K, Ca) analysed in thin-sample mode tended to yield underestimated concentrations. In contrast, heavier elements (e.g., Fe, Mn, Ni) showed overestimated values, which can be attributed to an over-correction of absorption effects when the sample had not fully reached the “infinitely thick” condition.

Sample pretreatment in traditional ICP-based technologies is often time-consuming, expensive, and complex, which limits their suitability for measuring large numbers of samples (Nawar et al. 2020). In contrast, XRF offers precision, accuracy, sensitivity, a wide dynamic linear range, high efficiency, and it typically is less labour intensive. Based on our experience, a single operator can efficiently handle the measurement and data analysis of 200 or more samples within a single day. Additionally, the double-layer method allows for reducing the sample amount to as low as 20 mg without data distortion, a threshold comparable to the lowest required by ICP-based methods (Hansen et al. 2009). Although MXRF assesses fewer elements than traditional ICP-based technologies, it offers significant efficiency advantages while maintaining accuracy. Therefore, MXRF is ideal for analysing plants in their early developmental stages, such as *A. thaliana* grown in agar-plate systems or small tissues affected by biotic or abiotic stress.

Researchers increasingly seek to determine whether newly developed techniques are equivalent to those currently in use. This question is typically addressed through method-comparison studies (Hanneman 2008). Previous comparisons of XRF with ICP-based digestion methods have highlighted substantial element-specific variation. For instance, in plants, Paltridge et al. (2012) reported strong correlations for Zn and Se (R^2^ > 0.95), but weaker correlations for Fe. In soils, McStay et al. (2022) observed moderate correlations for Mn, Cu, Zn, and Pb (R^2^ = 0.41–0.73), while Ca, K, and P exhibited weak or non-significant correlations (R^2^ < 0.25). These discrepancies are likely attributable to matrix effects, as higher soil organic matter increases X-ray attenuation and Compton scattering, thereby reducing accuracy and precision. In this study, we compared MXRF and ICP-AES data from 144 plant samples. Among the 12 analysed elements, 11 exhibited strong linear correlations between the two techniques, whereas Se showed poor agreement. This is consistent with known analytical challenges, as Se detection by ICP-AES is hindered by its high ionization potential, which reduces excitation efficiency and results in low sensitivity and high detection limits (Al-Hakkani 2019). Moreover, the Se concentrations in our samples were generally low (< 10 mg·kg^−1^), further contributing to the weak correlation. A further point of comparison lies between portable and desktop XRF systems. Portable/handheld XRF instruments have been widely evaluated and reviewed, demonstrating utility in rapid screening, non-destructive, and in situ analyses (van der Ent et al. 2019). However, their application to biological matrices is limited by strong scattering and matrix effects. For instance, Pessanha et al. (2009) reported that high background noise hinders the characterization of biological samples and paper documents using portable systems. In contrast, desktop (enclosed) instruments, such as the MXRF instrument tested here, employ monochromatic excitation, which reduces background noise and enhances quantification. Moreover, portable XRF are ‘open’ systems with a much lower power X-ray tube (5 Watt source) compared to 'closed' desktop systems, like the JP500 (which has a 20-Watt source).

Overall, this study established a standard method for quantifying 12 elements in plant samples using a new type of MXRF spectrometer. The optimized detection duration was set to 30 s, and the sample mass was 20 mg for single-layer film mode and 100 mg for double-layer film mode. The FP calibration combined with validation of large-scale empirical sample using ICP-AES confirmed the reliability of this method. The characteristics of the MXRF technique will help researchers conduct plant science research more efficiently in future.

## Supplementary Information

Below is the link to the electronic supplementary material.

Supplementary file1 (DOCX 25 KB)

## Data Availability

The data that support this study will be shared upon reasonable request to the corresponding author.
