# Supplementary material for: Monochromatic X-ray fluorescence spectroscopy for major and trace element analysis in plant science applications
Source: Plant Soil. 2025 Nov 13;518(1):105–20. doi: 10.1007/s11104-025-07984-5 (PMC12830464; doi:10.1007/s11104-025-07984-5)
Supplement: Supplementary file 1 — Supplementary file1 (DOCX 25 KB) [file 11104_2025_7984_MOESM1_ESM.docx]

**SUPPLEMENTARY INFORMATION**

**Monochromatic X-ray fluorescence spectroscopy for major and trace element analysis in plant science applications**

Chenyu Zhang^1^, Lucas Charrois^2^, Julien Jacquet^3^, Catherine Sirguey^2^,

Zewu Chen^4^, Antony van der Ent^1,2,3*^

^1^Laboratory of Genetics, Wageningen University and Research, The Netherlands.

^2^Université de Lorraine, INRAE, LSE, F-54000, Nancy, France.

^3^Econick SAS, Lunéville, France.

^4^Z-Spec Inc., Unites States.

*Corresponding author: antony.vanderent@wur.nl

**Table S1.** Preparation of multi-element standard solutions for calibration

| **Element** | **Atomic mass** | **Component** | **Molecular weight (g mol^-1^)** | **Weight (g)** | **Volume (mL)** | **Element concentration (g L^-1^)** |
| --- | --- | --- | --- | --- | --- | --- |
| As | 74.92 | Sodium arsenate dibasic heptahydrate | 312.01 | 2.08 | 10 | 50 |
| Ca | 40.08 | Calcium chloride dihydrate | 147.01 | 1.83 | 10 | 50 |
| Co | 58.93 | Cobalt chloride hexahydrate | 237.93 | 2.02 | 10 | 50 |
| Cu | 63.55 | Copper(II) sulfate pentahydrate | 249.69 | 1.96 | 10 | 50 |
| Fe | 55.85 | Iron(II) sulfate heptahydrate | 278.01 | 2.49 | 10 | 50 |
| K | 39.1 | Potassium Chloride | 74.55 | 0.95 | 10 | 50 |
| Mn | 54.94 | Manganese(II) sulfate tetrahydrate | 223.06 | 2.03 | 10 | 50 |
| Ni | 58.7 | Nickel chloride hexahydrate | 237.69 | 2.02 | 10 | 50 |
| Pb | 207.2 | Lead acetate trihydrate | 379.33 | 0.92 | 10 | 50 |
| Se | 78.96 | Sodium selenate | 188.94 | 1.20 | 10 | 50 |
| TI | 204.38 | Thallium nitrate | 266.39 | 0.65 | 10 | 50 |
| Zn | 65.38 | Zinc acetate dihydrate | 219.51 | 1.68 | 10 | 50 |

**Table S2.** Preparation of spiked samples for calibration

| **Stock concentration (g L^-1^)** | **Volume (μL)** | **Substrate weight (g)** | **Final concentration (mg kg^-1^)** |
| --- | --- | --- | --- |
| 0.0025 | 400 | 1 | 1 |
| 0.025 | 200 | 1 | 5 |
| 0.025 | 400 | 1 | 10 |
| 0.25 | 200 | 1 | 50 |
| 0.25 | 400 | 1 | 100 |
| 2.5 | 200 | 1 | 500 |
| 25 | 40 | 1 | 1000 |
| 25 | 200 | 1 | 5000 |
| 25 | 400 | 1 | 10000 |

**Table S3.** ANOVA for thin samples.

| **Source** | **SS** | **df** | **MS** | **F-value** | **Significance** |
| --- | --- | --- | --- | --- | --- |
| A | 7407.13 | 2.00 | 3703.57 | 104.99 | 0.00 |
| B | 46.83 | 2.00 | 23.42 | 0.66 | 0.56 |
| Error | 141.11 | 4.00 | 35.28 |  |  |
| Total | 32516.96 | 9.00 |  |  |  |

**Table S4.** ANOVA for thick samples.

| **Source** | ***SS*** | ***df*** | ***MS*** | ***F*-value** | **Significance** |
| --- | --- | --- | --- | --- | --- |
| A | 312.91 | 2.00 | 156.46 | 1.24 | 0.38 |
| B | 63.62 | 2.00 | 31.81 | 0.25 | 0.79 |
| Error | 505.19 | 4.00 | 126.30 |  |  |
| Total | 88418.80 | 9.00 |  |  |  |

**Table S5.** Range analysis for thin samples.

| **Level** | **A** | **B** |
| --- | --- | --- |
| *K_1_* | 51.75 | 166.05 |
| *K_2_* | 159.3 | 149.3 |
| *K_3_* | 262.55 | 158.25 |
| *k_1_* | 17.25 | 55.35 |
| *k_2_* | 53.1 | 49.77 |
| *k_3_* | 87.52 | 52.75 |
| *R* | 70.27 | 5.58 |

**Table S6.** Range analysis for thick samples.

| **Level** | **A** | **B** |
| --- | --- | --- |
| *K_1_* | 318.96 | 302.24 |
| *K_2_* | 292.65 | 284.62 |
| *K_3_* | 275.99 | 300.74 |
| *k_1_* | 106.32 | 100.75 |
| *k_2_* | 97.55 | 94.87 |
| *k_3_* | 92 | 100.25 |
| *R* | 14.32 | 5.37 |
